# Evaluation of Circulating Proteins and Hemodynamics Towards Predicting Mortality in Children with Pulmonary Arterial Hypertension

**DOI:** 10.1371/journal.pone.0080235

**Published:** 2013-11-20

**Authors:** Brandie D. Wagner, Shinichi Takatsuki, Frank J. Accurso, David Dunbar Ivy

**Affiliations:** 1 Department of Biostatistics and Informatics, Colorado School of Public Health, University of Colorado Denver, Aurora, Colorado, United States of America; 2 Department of Pediatrics, University of Colorado School of Medicine, Children's Hospital Colorado, Aurora, Colorado, United States of America; Keio University School of Medicine, Japan

## Abstract

**Background:**

Although many predictors have been evaluated, a set of strong independent prognostic mortality indicators has not been established in children with pediatric pulmonary arterial hypertension (PAH). The aim of this study was to identify a combination of clinical and molecular predictors of survival in PAH.

**Methods:**

This single-center, retrospective cohort study was performed from children with PAH between 2001 and 2008 at Children's Hospital Colorado. Blood samples from 83 patients (median age of 8.3 years-old) were obtained. We retrospectively analyzed 46 variables, which included 27 circulating proteins, 7 demographic variables and 12 hemodynamic and echocardiographic variables for establishing the best predictors of mortality. A data mining approach was utilized to evaluate predictor variables and to uncover complex data structures while performing variable selection in high dimensional problems.

**Results:**

Thirteen children (16%) died during follow-up (median; 3.1 years) and survival rates from time of sample collection at 1 year, 3 years and 5 years were 95%, 85% and 79%, respectively. A subset of potentially informative predictors were identified, the top four are listed here in order of importance: Tissue inhibitors of metalloproteinases-1 (TIMP-1), apolipoprotein-AI, RV/LV diastolic dimension ratio and age at diagnosis. In univariate analysis, TIMP-1 and apolipoprotein-AI had significant association with survival time (hazard ratio [95% confidence interval]: 1.25 [1.03, 1.51] and 0.70 [0.54–0.90], respectively). Patients grouped by TIMP-1 and apolipoprotein-AI values had significantly different survival risks (p<0.01).

**Conclusion:**

Important predictors of mortality were identified from a large number of circulating proteins and clinical markers in this cohort. If confirmed in other populations, measurement of a subset of these predictors could aid in management of pediatric PAH by identifying patients at risk for death. These findings also further support a role for the clinical utility of measuring circulating proteins.

## Introduction

Pulmonary arterial hypertension (PAH) is a progressive pulmonary vascular disease, characterized by remodeling of small to medium-sized pulmonary arteries, leading to elevated pulmonary vascular resistance and ultimately to progressive right heart failure and death [1]. Recent observational studies have identified predictors of survival in adult patients with PAH, including New York Heart Association functional class, six-minute walk distance, maximal oxygen consumption, echocardiographic data such as myocardial performance index, tricuspid annular plane systolic excursion, hemodynamic characteristics, and response to vasodilators [2]–[10]. However, the demographics of children are different than adults and prognostic indicators in pediatric patients with PAH are not well understood [11]–[21].

Recently, inflammatory processes have been highlighted in various types of PAH and circulating inflammatory proteins and progenitor cells may play a significant role in the development of PAH. Evidence of inflammation in the PAH population is supported by production of proinflammatory cytokines, including interleukins (IL) [5], [10], growth factors including platelet-derived growth factor [22]–[23], vascular endothelial growth factor [24], and chemokines. Further, progenitor cells, such as fibrocytes and myeloid derived suppressor cells, may contribute to the fibroproliferative changes that characterize pulmonary hypertensive vasculopathy [25]–[26]. Although these proteins are associated with different pathogenic mechanisms and have involvement of different tissues, their combined involvement eventually contributes to the development of PAH, including endothelial and vascular smooth muscle cell dysfunction and proliferation in pulmonary vasculature. Importantly, each of these molecular markers has demonstrated clinical utility, particularly as predictors of mortality in adult patients with PAH. Although B type natriuretic peptide (BNP) and N-terminal pro-B type natriuretic peptide (NTproBNP) have also been established as predictors in pediatric patients with PAH [27]–[29], current identified molecular markers, such as growth factors and proteases have not been evaluated in pediatric populations. Hemodynamic parameters have also been shown to correlate with prognosis in children with PAH. Pulmonary artery capacitance index (PACi) identified as a new predictor of mortality in adult and pediatric patients with PAH, reflects the ability of the pulmonary artery to dilate with each contraction of the right ventricle [30]–[32]. Therefore, despite having many variables evaluated individually, a set of strong independent prognostic indicators has not been established in pediatric populations. The aim of this study was to determine which circulating proteins and/or clinical variables, such as hemodynamics, can provide strong prognostic information in the management of children with PAH.

## Methods

### Ethics Statement

The study was approved by the Colorado Multiple Institutional Review Board (COMIRB). Written informed consent and HIPPA Authorization were obtained from all patients over the age of 17 years or from parents or legal guardians of participants younger than 18 years. Assent was obtained from all participants 7 years of age and older.

### Study Subjects

This single-center, retrospective cohort study was performed using clinical data from children with PAH between May 2001 and October 2008 at Children’s Hospital Colorado. Patients included in this study had diagnoses of idiopathic - primary pulmonary hypertension or congenital heart disease and had a banked blood sample available. All patients were being treated with appropriate therapies to manage their PAH during sample collection [33].

### Clinical Data

Exercise capacity was assessed by 6-minute walk distance during follow-up. We measured peak oxygen consumption during the cardiopulmonary test and calculated a ratio of minute ventilation to carbon dioxide output as ventilatory efficiency. Six-minute walk distance was most often performed the day before the catheterization; however, measurements from within 90 days of the blood draw were included. Right ventricular (RV) and left ventricular (LV) diastolic dimension are standard measurements by m-mode echocardiography [34]. The ratio of RV over LV dimension aims to compare RV to LV size and was available for all patients.

By right heart catheterization using a flow-directed Swan-Ganz catheter and a systemic arterial line for monitoring, we measured mean right atrial pressure (mRAP), mean pulmonary artery pressure (mPAP), mean systemic blood pressure, and pulmonary capillary wedge pressure (PCWP). Accordingly, we calculated pulmonary vascular resistance index (PVRI) and pulmonary vascular resistance/systemic vascular resistance ratio. Cardiac output was obtained using thermo-dilution and cardiac index (CI) was calculated. If a significant intra-cardiac defect remained, cardiac output was obtained by the Fick method using the LaFarge estimation [35]. PAC was calculated as stroke volume divided by the change in pulmonary artery pressure (pulse pressure) [30]. PACi to body surface area was evaluated using catheterization data.

### Measurements of Circulating Proteins

Blood samples were obtained from the femoral vein during right heart catheterization, but in rare instances were instead collected using venipuncture within 5 days of the procedure. Twenty-seven protein levels were measured in the blood samples (Table 1). Circulating proteins investigated included inflammatory cytokines, growth factors, and vasoactive proteins, measured using 6 different assays, which included a mix of enzyme-linked immunosorbent and bead-based assays. Further details of these assays are included in file S1.

**Table 1:** List of investigated biomarkers.

Molecule of cell proliferation and vascular remodeling	
Growth factors	VEGF, PDGF-AA, PDGF-AB/BB, EGF, FGF2, TGF-β
Protease	MMP-9, TIMP-1
Tyrosine kinase	FLT-3
Inflammatory cytokines and molecules:	
IL-1β, IL-2, IL-6, IL-8, IL-10, TNF- α, E-selectin, ICAM-1, VCAM-1, PAI-1	
Vasoactive proteins:	
ET-1, BNP, NTproBNP	
Other markers:	
Hemoglobin, Bilirubin, Transaminase, Creatinine, Uric acid,Apolipoprotein A-1, C-2, C-3	

BNP; brain natriuretic peptide, EGF; Epidermal growth factor, ET-1; Endothelin-1, FGF2; Fibroblast growth factors 2, FLT3; fms-like tyrosine kinase receptor 3, ICAM-1 Intercellular Adhesion Molecule 1, IL; Interleukin, MMP-9; Matrix metallopeptidase 9, NTproBNP; N-terminal pro-B-type natriuretic peptide, PAI-1 Plasminogen activator inhibitor-1, PDGF; platelet-derived growth factor, TGF-β; Transforming growth factor-β, TIMP1; metallopeptidase inhibitor 1, TNF-α; Tumor necrosis factor-α, VCAM-1 vascular cell adhesion molecule 1, VEGF; Vascular endothelial growth factor.

### Statistical Analyses

The limit of detection value for each assay was used for those values reported below this limit. Descriptive statistics were calculated using medians and inter-quartile range (IQR) for continuous variables and percentages for categorical variables. The outcome used for analyses was all-cause mortality. Cox survival models were used to assess univariate associations between each marker and survival performed using SAS version 9.3 software (SAS Institute Inc., Cary, NC, 2011). Random survival forests (RSF) are a popular method commonly employed in problems where the numbers of predictors outnumber a small number of events and have been shown to have good statistical properties when a large number of predictors are present. [36] RSF were used to evaluate all variables in a multivariate fashion. RSF can uncover complex data structures while performing variable selection in high dimensional problems. The mechanisms and pathways of PH are complex and likely include interactions between protein biomarkers. Random forests are capable of handling these complex data structures while having fewer restrictive assumptions compared to a Cox survival model. We used RSF here to identify important predictors of survival in pediatric PAH, a subset of which could then be used to generate a Cox model in a larger cohort. A survival forest of 5000 trees was implemented using the randomSurvivalForest R-package. Specifics of the other parameters used for the RSF approach are included in the supplementary material (File S1). Maximal subtrees were investigated to determine a variable’s predictive ability [37].

## Results

### Patient Demographics

**Figure 1:**
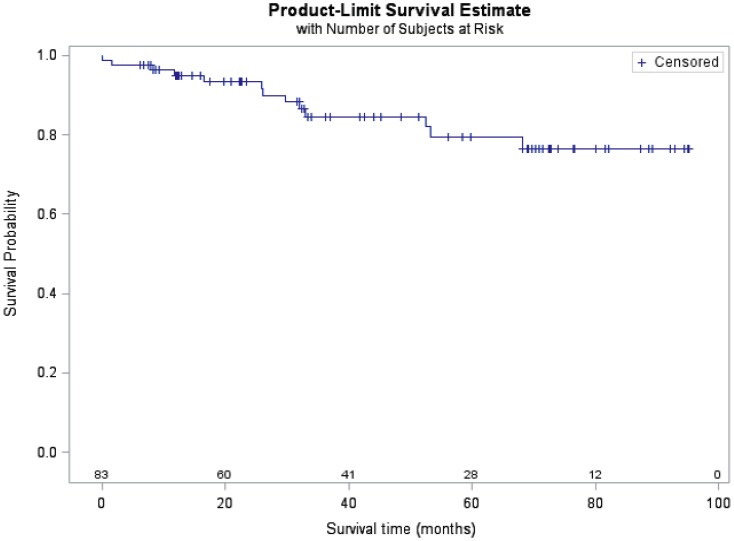
Survival curve for all children with pulmonary arterial hypertension. Kaplan-Meier curve displaying the overall survival in the studied cohort. The number of subjects at-risk are displayed along the x-axis.

This study included 83 PAH children with a median age of 8.3 years old (IQR 4–13.8 years-old) with 41(49%) female patients and average growth (Table 2). The different etiologies of PAH included: idiopathic PAH (n = 36) and PAH associated with congenital heart disease (n = 47), specific diagnoses are provided in the online supplement. The hemodynamic data suggests significant pulmonary hypertension in this cohort. Patients were followed for 304 person years and thirteen children (15.7%) died during follow-up. Survival rates from time of sample collection at 1 year, 3 years and 5 years were 95%, 85% and 79%, respectively (Figure 1).

**Table 2:** Patient demographics and clinical measurements.

Patient demographics	
Number	83
Age (years) – median (IQR)	8.3 (3.9–13.8)
Age at diagnosis (years)	4.2 (0.8–7.4)
Female	41 (49%)
BMI percentile	51.1 (20.4–79.7)
Idiopathic pulmonary arterial hypertension	36 (43%)
**Clinical measurements** median (IQR)	
6 minute walk distance (meters) (n = 36)	479 (413–564)
Right heart catheterization (n = 67)	
*Pulmonary artery pressure (mm Hg)*	40 (27–62)
*Pulmonary capillary wedge pressure (mm Hg)*	8 (6–9)
*Right atrial Pressure (mmHg)*	6 (4–7)
*Pulmonary vascular resistance index (unitxm^2^)*	7.9 (4.6–15.5)
*Pulmonary/systemic vascular resistance ratio*	0.73 (0.46–0.94)
*Cardiac Index (L/min/m^2^)*	4.0 (3.0–4.8)
*RV/LV*	0.6 (0.5–0.8)
**Survival outcomes**	
Follow-up (months) – median (IQR)	37.0 (16.5–70.3)
Expired	13 (15.7%)

IQR; inter-quartile range.

### Description of Protein Levels

All the proteins measured, with the exception of Interleukin-1 beta, were detected in greater than 50% of samples. A description of all the circulating protein levels is included in Table S2 in File S1. These molecular markers were further evaluated to assess whether an association with demographic factors exist and to establish which proteins are highly correlated with each other. There were significant associations with demographic factors, especially age, for several of the proteins which could result in confounding effects when conducting future studies of these proteins in similar populations (Figure S1 in File S1).

### Univariate Association with Survival

**Figure 2:**
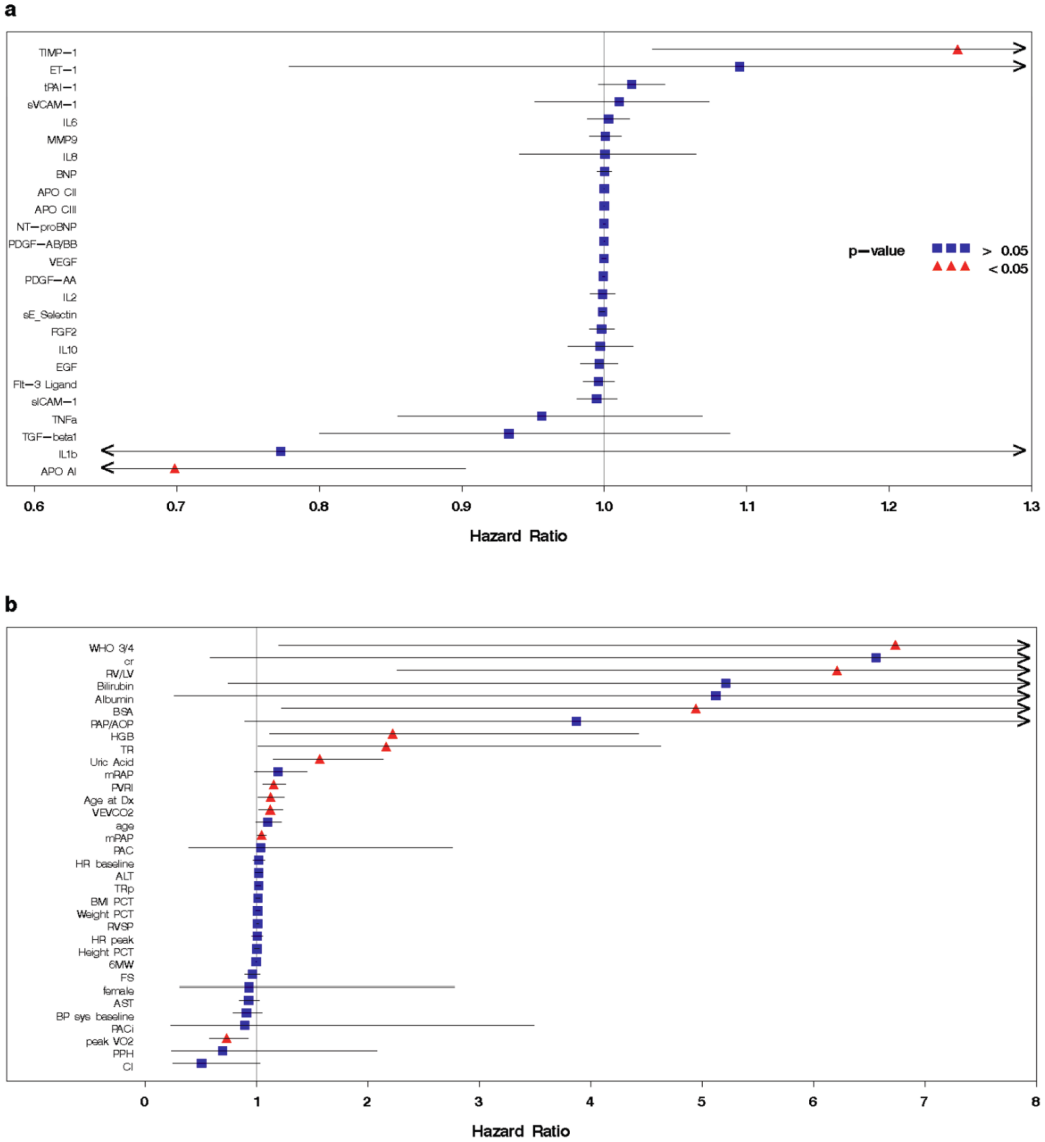
Univariate Cox regression results. Hazard ratios (points) and corresponding 95% confidence intervals (bars) for circulating proteins (a) and clinical variables (b). Variables with confidence intervals not including 1.0 are indicated with a red triangle.

In total, there were 46 predictors analyzed, which included 27 circulating proteins, 7 demographic variables and 12 hemodynamic and echocardiographic variables. The most predictive molecular markers when investigated in a univariate manner, were uric acid (Hazard ratio (HR) [95%Confidence interval]: 1.6 [1.2,2.1] per 1 mg/dl), APO-AI (0.70 [0.54, 0.90] per 100 µg/ml), TIMP-1 (1.25 [1.03, 1.51] per 10 ng/ml), and hemoglobin (2.2 [1.1, 4.4] per 1 g/dl), whereas the most predictive clinical markers were RV/LV diastolic dimension ratio (6.2 [2.3, 17.1]), pulmonary vascular resistance index (1.15 [1.06, 1.26]), peak oxygen consumption (0.73 [0.58, 0.93]), VE/VCO2 slope (1.13 [1.02, 1.24]), pulmonary artery pressure (1.05 [1.01, 1.09]), age at diagnosis (1.13 [1.02, 1.24]), body surface area (4.94 [1.23, 19.9]), and WHO FC (6.73 [1.2, >20] (Figure 2). In this study, PACi was not significantly associated with survival (p = 0.88).

### Identification of Survival Predictors

**Figure 3:**
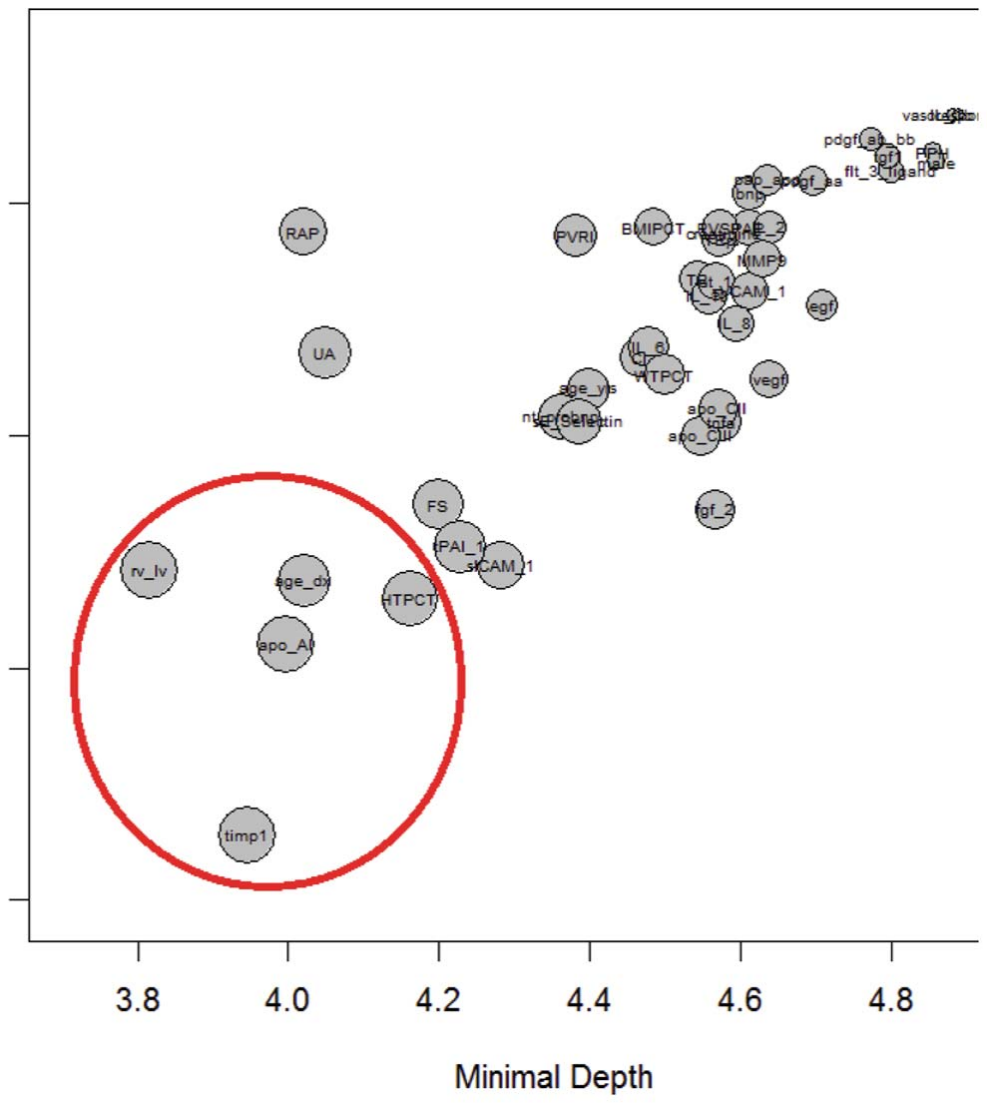
RSF maximal subtree results. Minimal depth versus distance of the second-closest maximal subtree. A circle’s diameter is proportional to the average number of maximal subtrees for a given variable. Predictive variables are displayed in the lower left corner with a larger radius. The top 5 predictors are indicated with a red circle.

**Figure 4:**
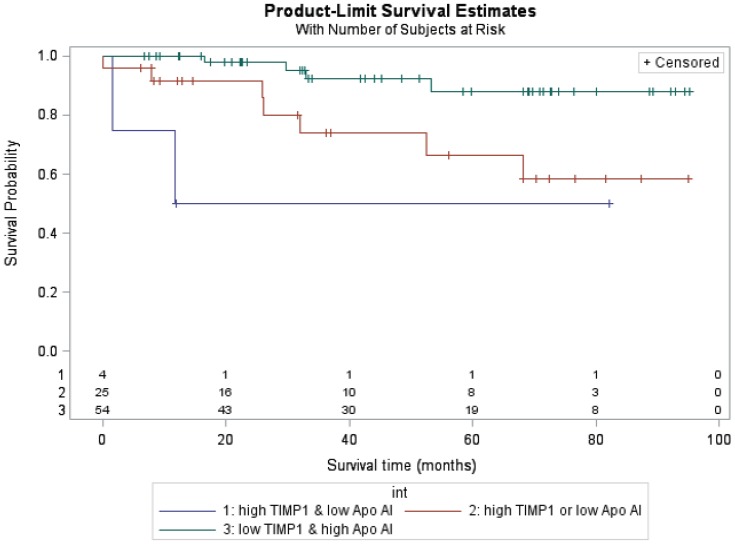
Survival curves comparing patients stratified by TIMP-1 and apolipoprotein-AI values. Patients with high TIMP-1 and low apolipoprotein-AI values (red) had lower survival (log-rank test p-value <0.01). The number of subjects at-risk are displayed along the x-axis.

Using the RSF approach, all 46 protein and clinical variables were included to identify those most associated with survival of PAH patients. A subset of potentially informative predictors were identified, the top five are listed here in order of importance: TIMP-1, apolipoprotein-AI, RV/LV, age at diagnosis and height percentile (Figure 3). These circulating markers were not significantly correlated with any other protein biomarkers and were relatively stable within patients (Figures S2 and S3 in File S1). In addition to estimating the relative importance of the variables, RSF is useful for investigating the functional form of the relationship between predictors and survival. For all top predictors, with the exception of apolipoprotein-AI, higher values were associated with increased risk (Figure S4 in File S1). For TIMP-1, this risk appears to be most prominent at values of 125 ng/ml or greater and for apolipoprotein-AI this risk increases when values are less than 1.2 mg/ml. Patients grouped using these cutoffs had significantly different survival risks (p<0.01). Of the 4 patients with higher TIMP-1 values (values >125 ng/ml) and lower apolipoprotein-AI (values <1.2 mg/ml), 50% died compared to 28% of patients with either a high TIMP-1 or a low apolipoprotein-AI and only 7% of patients in the lower risk group (Figure 4).

## Discussion

This is the first investigation in a relatively large pediatric pulmonary hypertension population investigating the role of almost 30 proteins combined with clinical and demographic variables towards predicting survival. Moreover, novel data mining techniques were utilized to identify the most informative predictors of survival in a complex disease to aid in furthering PAH proteomic research. TIMP-1, an inhibitor of matrix metalloproteinases, was identified as an important predictor in both the univariate and multivariate approaches. In addition, apolipoprotein-A1 was identified along with other currently known informative variables such as, RV/LV, age at diagnosis, height and uric acid. This investigation offers insight into which variables would be useful to collect in future studies as well as providing further evidence for the role of matrix metalloproteinases and the extracellular matrix in PAH.

Previous work suggests that an imbalance of TIMP-1 and matrix metalloproteinases may play a role in severe pulmonary vascular remodeling in pulmonary hypertension [38]–[40]. TIMP-1 has been shown to be over-expressed by proinflammatory cytokines and pro-fibrotic stimuli in a failing heart; this over-expression promotes cell proliferation and may have anti-apoptotic effects [41]. Excessive TIMP-1 is also associated with fibrotic change, such as pulmonary fibrosis [42]. It has recently been shown that pulmonary vasodilators may decrease matrix metalloproteinase production and contribute to vascular remodeling[43]–[44].

Although the survival rate has been dramatically improved with targeted pulmonary vasodilators in pediatric patients with PAH, 5-year estimated survival rate is still estimated at 74% [13]. Previous studies have demonstrated that circulating proteins, echocardiographic data, and hemodynamics have predictive value for prognosis in pediatric patients with PAH [12]–[13], [15]–[21], [45]. Although many predictive factors associated with worse outcomes have been evaluated in the pediatric population, there is limited information comparing many factors including both protein levels and hemodynamics in children with PAH. One such study reported that higher functional class, higher pulmonary/systemic arterial pressure ratio, lower cardiac index, and higher NTproBNP and uric acid were associated with decreased survival [20]. In a previous study poor height and weight at presentation predicted worse survival [18]. It is unclear why taller children had worse prognosis in our study, but this may be due to them being older at diagnosis.

Similarities are observed between the predictors in children and adults with PAH. In a current large adult registry, PAH associated with connective tissue disease, higher functional class, lower six-minute walk distance, higher right arterial pressure, lower systemic blood pressure, higher heart rate, worse pulmonary function test, higher BNP, renal insufficiency, and pericardial effusion all predicted mortality [2], [46]. The traditional clinical endpoints used in adults (i.e., functional class and six-minute walk distance) are difficult to assess in children, highlighting the need for pediatric specific measures. Although noninvasive tests are useful in pediatric PAH, cardiac catheterization still remains the gold standard for evaluating the disease severity and treatment responses in children. Many previous studies have demonstrated that pulmonary artery pressure and/or pulmonary vascular resistance index were prognostic indicators for children with PAH. The compliance of the pulmonary artery measures how much the pulmonary vasculature will dilate with each contraction of the right ventricle [31]. Currently, PACi, an indicator of the workload on the right ventricle as a total compliance of the pulmonary vascular bed, has been evaluated for predicting survival in pediatric PAH patients [32]. However, we found PACi did not have superiority for predicting mortality compared to other commonly collected measures. It is possible that a measure of total right ventricular afterload, pulmonary vascular input impedance [47], may be a good predictor of survival, but we were unable to measure impedance in all patients.

Several limitations should be mentioned. First, our study was an observational cohort study from a single center (at moderate altitude) with a small number of events. Therefore, clinical findings regarding the prognostic impact of predictors remain to be confirmed in a larger multi-site study. Second, children were individually treated with various vasodilator therapies according to their conditions. The variety of treatments might be potentially associated with their prognosis. Additionally, patients with complex heart disease can have other cardiovascular risk factors on the risk of mortality which may not be captured in this study.

In conclusion, using a novel data mining approach we identified potentially important predictors of mortality in this cohort. If confirmed in other populations, measurement of a subset of these predictors could aid in management of pediatric PAH by identifying patients at risk for death. These findings also further support a role for the clinical utility of measuring circulating proteins. Higher TIMP-1 was associated with mortality in pediatric patients with PAH and was the best predictor among various clinical data.

## Supporting Information

File S1 **This contains Figures S1–S4 and Tables S1 and S2. Figure S1. Test statistics evaluating the association between molecular markers and demographic variables. Figure S2. Heatmap displaying the magnitude of the correlation between measured circulating proteins. Figure S3. The points correspond to the intraclass correlation coefficient (ICC) for each molecular marker listed and the lines denote the 95% confidence intervals of the estimates.** The higher the ICC value, the more similar the protein levels were within a patient. **Figure S4. Functional form of top predictor variables. Plots for top 4 predictors from RSF are displayed. Table S1. Detailed Diagnosis of heart disease diagnosis in congenital heart disease patients. Table S2. Description of circulating protein levels in pediatric PH patients.**
(DOCX)
