# Supplementary material for: Evaluation of Circulating Proteins and Hemodynamics Towards Predicting Mortality in Children with Pulmonary Arterial Hypertension
Source: PLoS One. 2013 Nov 20;8(11):e80235. doi: 10.1371/journal.pone.0080235 (PMC3835871; doi:10.1371/journal.pone.0080235)
Supplement: File S1 — This contains Figures S1–S4 and Tables S1 and S2. Figure S1. Test statistics evaluating the association between molecular markers and demographic variables. Figure S2. Heatmap displaying the magnitude of the correlation between measured circulating proteins. Figure S3. The points correspond to the intraclass correlation coefficient (ICC) for each molecular marker listed and the lines denote the 95% confidence intervals of the estimates. The higher the ICC value, the more similar the protein levels were within a patient. Figure S4. Functional form of top predictor variables. Plots for top 4 predictors from RSF are displayed. Table S1. Detailed Diagnosis of heart disease diagnosis in congenital heart disease patients. Table S2. Description of circulating protein levels in pediatric PH patients. (DOCX) [file pone.0080235.s001.docx]

**ONLINE SUPPLEMENT**

Details of protein assays

1. Human Cardiovascular Disease Panel 1; plasminogen activator inhibitor-1 (PAI-1), E-selectin, intercellular adhesion molecule 1 (ICAM-1), vascular cell adhesion molecule
   1 (VCAM-1)

The Human Cardiovascular Disease Panel 1 multiplex bead-based kit is manufactured by LINCO Research, a division of Millipore Inc. (St Charles, MO, USA). Blood sample was measured using a Luminex® 200™ instrument (Bio-Plex 200 System Instrument, Bio-Rad Laboratories, Inc. CA, USA) and the data was analyzed using Bio-Plex Manager software version 4.1.1.

1. Human Cardiovascular Disease Panel 3; IL-1β, IL-6, IL-8, IL-10, NTproBNP, Tumor necrosis factor-α (TNF-α)

The Human Cardiovascular Disease Panel 3 multiplex bead-based kit is manufactured and distributed by Millipore.

1. Human Milliplex Panel; IL-2, Epidermal growth factor (EGF), Fibroblast growth factors 2 (FGF-2), ms-like tyrosine kinase receptor 3 (Flt-3) ligand, VEGF PDGF-AA, PDGF-AB/BB

The Human Milliplex Panel is a multiplex bead-based kit that is manufactured by LINCO Research and distributed by Millipore.

1. Human Apolipoprotein (Apo) Panel; Apo AI, ApoCII, ApoCIII

The Human Apolipoprotein Panel multiplex bead-based kit is manufactured by LINCO Research and distributed by Millipore.

1. Human Beadlyte Transforming growth factor-β (TGF-β) panel; TGFβ1, TGFβ2, TGFβ3

The TGF-β multiplex bead-based kit is manufactured by Upstate and distributed by Millipore.

1. Enzyme-linked immunosorbent assays; tissue inhibitor of mealloproteinases (TIMP-1), matrix metalloproteinase (MMP-9), endothelin-1 (ET-1), BNP

The TIMP-1 kit, MMP-9 kit, and ET-1 kit are Enzyme-linked immunosorbent assays manufactured and distributed by R&D Systems (Roche Diagnostics Inc., IN, USA). Plasma BNP was assayed on i-STAT® system using the two-site enzyme-linked immunosorbant assay (Abbott Laboratories, IL, USA).

Description of random survival forest

Random survival forests (RSF) were used to further evaluate all variables. A survival forest of 5000 trees was implemented using the randomSurvivalForest R-package. Fifteen candidate variables were selected for each node. Random log-rank splitting with an “nsplit” value of 10 was used. Trees were grown by choosing a maximum of nsplit split points randomly for each candidate variable when splitting a node. Logrank splitting was applied to these random split points, and the node was split using the variable whose random split point maximized the log-rank statistic. The RSF analysis was used to select the top predictors and suggest cut-offs for each one using the approach described by Ishwaran *et al* [1]. The minimal depth is the distance from the root node to the root of the closest maximal *v*-subtree for a given *v*. It measures how far a case travels down the tree before encountering the first split on *v*, and indicates the predictiveness of *v*. The smaller the minimal depth, the greater the impact *v* has on prediction. If the minimal depth is 0, then *v* splits the root node, and the maximal *v*-subtree is the entire tree itself. A bubble plot of the distance of the second-closest maximal subtree (second-order depth) versus the distance of the closest maximal subtree (minimal depth) with a circle’s diameter proportional to the average number of maximal subtrees for a given variable can be useful for identifying predictive variables [1]. Those variable with the best predictive ability will be displayed in the lower left of the plot.

Comparisons of a Cox model and RSF have shown that both approaches have similar error rates. The Cox model gives a clinically understandable output on covariate impact, whereas RSF becomes more a "black box". However, RSF complements the Cox model by giving more insight and confidence toward relative importance of the model covariates. We used it here to identify important predictors of survival in pediatric PH, a subset of which could then be used to generate a Cox model in a larger cohort.

Diagnosis of Heart Disease in Congenital Heart Disease Patients

Of the 15 unrepaired CHD patients 5 had Eisenmenger syndrome

**Table S1.** Detailed Diagnosis of heart disease diagnosis in congenital heart disease patients

|  | Un-repaired | Repaired |
| --- | --- | --- |
| Atrial septal defect | 6 | 7 |
| Ventricular septal defect | 3 | 4 |
| Isolated pulmonary artery of ductal origin, "Absent PA" | 4 | 1 |
| Patent ductus arteriosus | 1 | 3 |
| Atrioventricular septal defect | 1 | 2 |
| Transposition of the great arteries | 0 | 3 |
| Atrial septal defect / Ventricular septal defect | 0 | 2 |
| Coarctation | 0 | 2 |
| Total anomalous pulmonary venous return | 0 | 2 |
| Atrial septal defect / Patent ductus arteriosus | 0 | 1 |
| Isolated pulmonary vein stenosis, s/p surgery | 0 | 1 |
| Double outlet right ventricle | 0 | 1 |
| Tetralogy of Fallot | 0 | 1 |
| Ventricular septal defect / Coarctation | 0 | 1 |
| Ventricular septal defect / Coarctation /Mitral stenosis | 0 | 1 |

Description of protein markers

**Table S2**. Description of circulating protein levels in pediatric PH patients

| Molecular function | Protein | N | Percent of samples with detectable values | Median (IQR) |
| --- | --- | --- | --- | --- |
| Cytokines | IL-1β  (pg/ml) | 83 | 6% | --- |
|  | IL-2  (pg/ml) | 81 | 51% | 3.2  (3.2, 16.4) |
|  | IL-6  (pg/ml) | 83 | 90% | 6.1  (1.7, 25.1) |
|  | IL-8  (pg/ml) | 83 | 98% | 3.4  (1.7, 6.8) |
|  | IL-10  (pg/ml) | 83 | 99% | 6.8  (3.7, 16.1) |
|  | TNF-α  (pg/ml) | 83 | 95% | 5.82  (2.96, 9.89) |
|  | Flt – 3 Ligand (pg/ml) | 81 | 58% | 9.0  (3.2, 46.1) |
| Growth Factors | TGF-β1  (ng/ml) | 68 | 82% | 6.89  (3.29, 12.66) |
|  | FGF-2  (pg/ml) | 81 | 93% | 27.0  (17.9, 44.6) |
|  | EGF  (pg/ml) | 81 | 81% | 25.8  (16.0, 64.3) |
|  | VEGF  (pg/ml) | 81 | 88% | 262.0  (105.9, 483.6) |
|  | PDGF-AA  (pg/ml) | 83 | 86% | 1066  (445, 1993) |
|  | PDGF – AB/BB (pg/ml) | 83 | 60% | 3281  (1600, 9068) |
| Binding | Apo AI  (ng/ml) | 83 | 100% | 1.33 x10^6^  (1.16 x10^6^, 1.47 x10^6^) |
|  | Apo CII  (ng/ml) | 83 | 99% | 3.36 x10^4^  (2.37 x10^4^, 5.33 x10^4^) |
|  | Apo CIII  (ng/ml) | 83 | 100% | 1.19 x10^5^  (0.87 x10^5^, 1.62x10^5^) |
|  | sE-Selectin (ng/ml) | 79 | 100% | 1215  (1014, 1445) |
|  | sICAM -1  (ng/ml) | 79 | 100% | 140.4  (111.9, 167.3) |
|  | sVCAM – 1  (ng/ml) | 79 | 100% | 12.17  (7.35, 18.88) |
| Protease inhibitors | PAI-1  (ng/ml) | 79 | 100% | 40.17  (31.70, 54.97) |
|  | TIMP-1  (ng/ml) | 80 | 95% | 87.89  (73.81, 109.89) |
| Protease | MMP-9  (ng/ml) | 80 | 96% | 72.5  (52.9, 115.4) |
| vasoactive | ET-1  (ng/ml) | 73 | 71% | 0.99  (0.47, 1.54) |
|  | NT-proBNP  (pg/ml) | 82 | 66% | 34.0  (16.0, 119.6) |
|  | BNP  (pg/ml) | 68 | 63% | 14.8  (5.8, 38.9) |
|  | Uric acid  (mg/dl) | 65 |  | 4.9  (3.9, 6.1) |
|  | Creatinine  (mg/dl) | 65 |  | 0.6  (0.5, 0.8) |

Supplementary Results

The identification of relevant demographic factors when studying each molecular marker was assessed using non-parametric rank-based tests. The proteins were compared across demographic variables which included age, gender and primary diagosis. Six proteins (IL-10, NTproBNP, SE selectin, SICAM1, TGFb1 and TNFa) were observed to have decreasing levels with age while two (APO CIII and VEGF) Increased with age (Figure S1). Two binding proteins, Apo CII and Apo CIII were significantly higher in PPH.


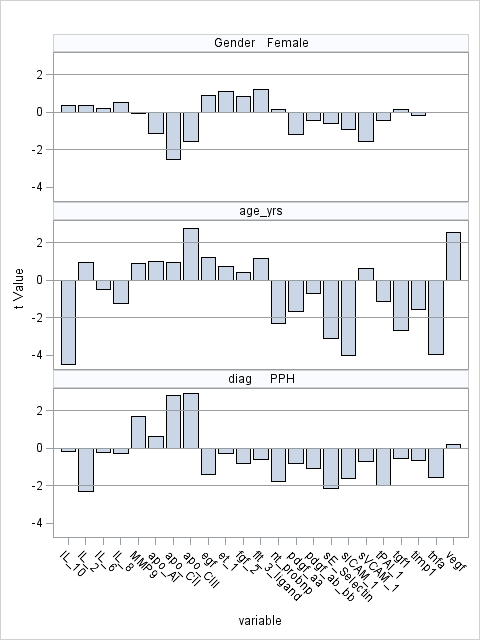


**Figure S1.** Test statistics evaluating the association between molecular markers and demographic variables. Positive t-values correspond to higher protein levels in females for Gender (top), with increasing age (middle), and in PPH for primary diagnosis (bottom). Values outside of the reference lines indicate statistical significance at 0.05.

The correlations between molecular markers were assessed to aid in the interpretation of the multivariate models. Markers which are highly correlated do not contribute orthogonal information resulting in one of the markers being less important in the prediction model. Spearman’s rank correlation coefficients were used to estimate the association between molecular markers. There were five pairs of proteins that were highly correlated indicating that inclusion of both in a future study would be unnecessary (Figure S2). These pairs include PDGF AA and PDGFAA/AB with TGFb1 and with each other, IL6 with IL8, and APO CIII with APO CII.


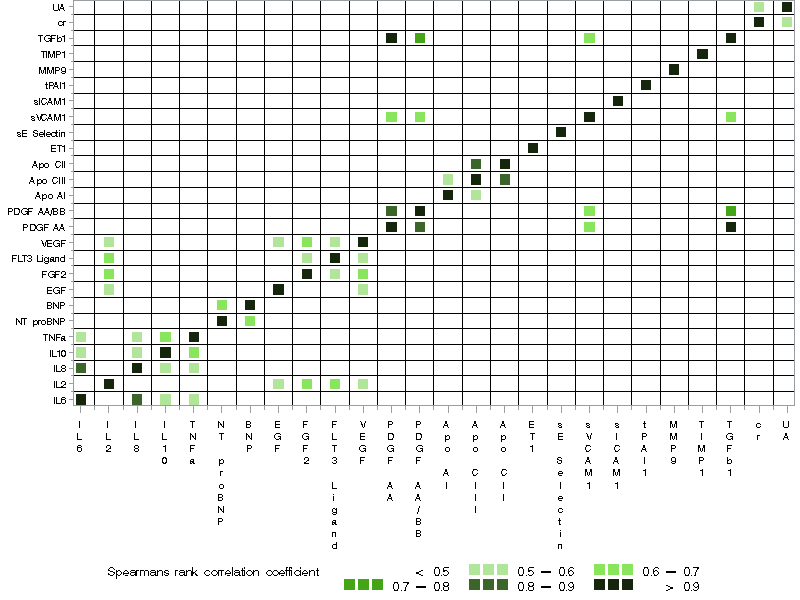


**Figure S2.** Heatmap displaying the magnitude of the correlation between measured circulating proteins.

A second sample was obtained in a subset (n = 39) of the 83 pediatric patients. These multiple measurements were used to estimate the intraclass correlation coefficient (ICC) for each molecular marker. This measure estimates the degree of homogeneity within patients. The ICC and corresponding 95% confidence intervals for each marker were estimated assuming a log-normal distribution (Figure S3).


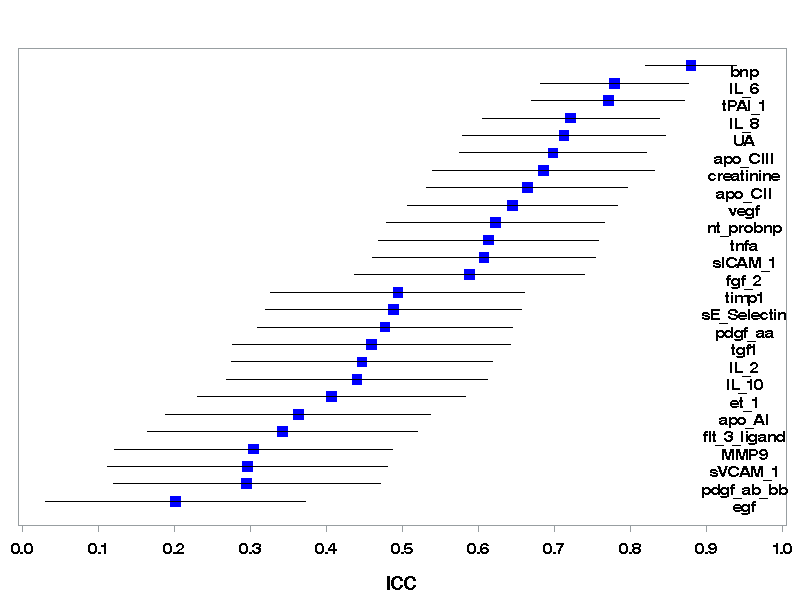


**Figure S3.** The points correspond to the intraclass correlation coefficient (ICC) for each molecular marker listed and the lines denote the 95% confidence intervals of the estimates. The higher the ICC value, the more similar the protein levels were within a patient.

In addition to estimating the relative importance of the variables, RSF is useful for investigating the functional form of the relationship between predictors and survival. For all top predictors, with the exception of apolipoprotein-AI, higher values were associated with increased risk (Figure S4). For TIMP-1, this risk appears to be most prominent at values of 125 ng/ml or greater and for apolipoprotein-AI this risk increases when values are less than 1.2 mg/ml.


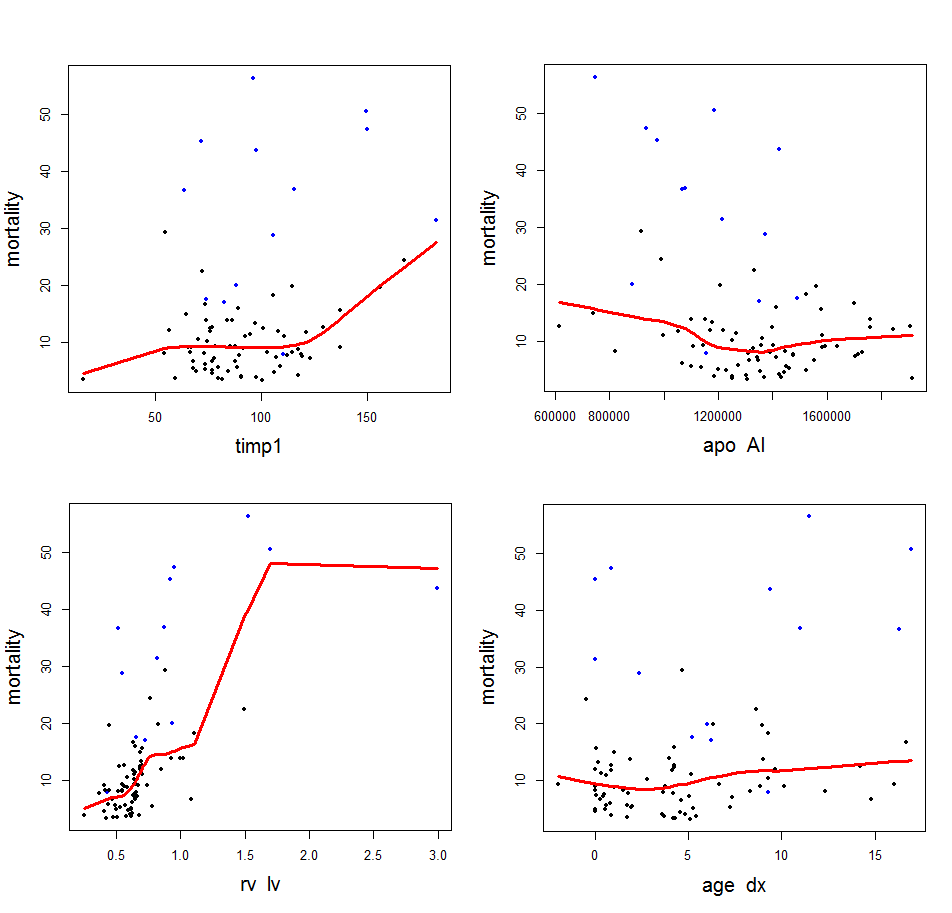


**Figure S4:** Functional form of top predictor variables. Plots for top 4 predictors from RSF are displayed. Values on the y-axis represent expected number of deaths for a given predictor, after adjusting for all other predictors. Events are represented in blue, censored observations are in black, the red line is a lowess curve displaying the functional form of the association.

References

1. Ishwaran H, Kogular UB, Gorodeski EZ, Minn AJ, Lauer MS (2010) High-Dimensional Variable Selection for Survival Data. Journal of American Statistical Association 105: 205-217.
